# High-Performance Thin-Layer Chromatography Hyphenated with Microchemical and Biochemical Derivatizations in Bioactivity Profiling of Marine Species

**DOI:** 10.3390/md17030148

**Published:** 2019-03-03

**Authors:** Snezana Agatonovic-Kustrin, Ella Kustrin, Vladimir Gegechkori, David W. Morton

**Affiliations:** 1 School of Pharmacy and Applied Science, La Trobe Institute for Molecular Sciences, La Trobe University, Edwards Rd, Bendigo 3550, Australia; d.morton@latrobe.edu.au; 2 Department of Pharmaceutical and Toxicological Chemistry named after Arzamastsev of the Institute of Pharmacy, I.M. Sechenov First Moscow State Medical University (Sechenov University), Moscow 119991, Russia; vgegechkori@gmail.com; 3 Department of Creative Arts and English, La Trobe University, Edwards Rd, Bendigo 3550, Australia; 20053085@students.latrobe.edu.au

**Keywords:** α-amylase inhibitors, antioxidants, cholinesterase inhibitors, effect-directed analysis (EDA), HPTLC, marine algae, phenolic lipids

## Abstract

Marine organisms produce an array of biologically active natural products, many of which have unique structures that have not been found in terrestrial organisms. Hence, marine algae provide a unique source of bioactive compounds. The present study investigated 19 marine algae and one seagrass collected from Torquay beach, Victoria, Australia. High-performance thin-layer chromatography (HPTLC) hyphenated with microchemical (DPPH•, p-anisaldehyde, and Fast Blue B) and biochemical (α-amylase and acetylcholine esterase (AChE) enzymatic) derivatizations was used to evaluate antioxidant activity, presence of phytosterols and phenolic lipids, α-amylase and AChE inhibitory activities of extract components. Significant α-amylase and AChE inhibitory activities were observed in samples 2, 6, 8 and 10. Antioxidant activities in the samples were found to be correlated to phytosterol content (R^2^ = 0.78), but was not found to be related to either α-amylase or AChE inhibitory activities. α-Amylase inhibitory activities were correlated to AChE inhibition (R^2^ = 0.77) and attributed to the phytosterol content, based on the similar peak position in the chromatograms with the β-sitosterol chromatogram. Samples 1, 8, and especially sample 20, were found to contain phenolic lipids (alkyl resorcinol derivatives) with significant antioxidant activities. The results suggest that these marine species have a significant number of bioactive compounds that warrant further investigation.

## 1. Introduction

Effect-directed analysis (EDA), based on the combination of chromatography with chemical and biochemical analyses, is widely used in drug discovery, especially in target-directed identification of biologically active molecules in complex samples [1,2,3]. Treatment of cardiovascular disorders, diabetes, dementia, and Alzheimer’s disease (AD) can be improved with drugs showing selective enzymatic inhibition/induction and antioxidant activity. The main purpose of diabetic therapies is to control the blood glucose level and to prevent complications such as cardiovascular disease. Diabetes is a chronic metabolic disease with high morbidity and mortality due to related complications. Uncontrolled diabetes leads to nephropathy, neuropathy, retinopathy, diabetic foot, ketoacidosis, increased risk of cardiovascular diseases, and hypertension [4]. Inhibition of starch digesting enzymes such as α-glucosidase and α-amylase delays absorption of glucose, lowers the risk of hypoglycaemia and provides greater benefits in preventing diabetic complications than conventional medications [5]. Drug inhibitors (starch blockers) such as acarbose and miglitol inhibit digestion of carbohydrates and decrease the intestinal absorption of glucose [5,6]. A more natural approach is to include functional foods in the diets of diabetic patients, from plants and marine organisms that are rich in α-glucosidase and/or α-amylase inhibitors. AD is an age-related neurodegenerative disease of the brain. The brain goes through a number of biological and chemical changes resulting in progressive atrophy of certain brain areas as the nerve cells die, brain inflammation, and cognitive impairment. Affected brain tissue shows two neuropathological marks: plaques and tangles. The formation of plaques and tangles is associated with the increased activity of the enzyme AChE, leading to decreased levels of neurotransmitter acetylcholine (ACh) and synaptic alterations [7]. Current treatments for AD are just palliative rather than curative. Anti-AD drugs approved by FDA are mostly based on inhibition of AChE and antagonism of the N-methyl-D-aspartate (NMDA) receptor [7]. These drugs are, however, associated with side-effects. Hence, pharmaceutical research aims at the discovery of new and more effective compounds with strong neuroprotective potential and fewer or no side-effects compared to synthetic drugs. According to recent studies, diabetes and AD are related. Findings show that insulin production in the brain declines as AD advances [8]. This demonstrates that AD is most likely a neuroendocrine disorder, or another type of diabetes.

Seaweeds or marine macroalgae are a renewable, living source of highly bioactive secondary metabolites with reported antioxidant [9] and α-amylase inhibitory activities [10]. Hence, there is great possibility that the isolation, characterization, and pharmacological study of marine algae can lead to the discovery of novel antidiabetic and anti-AChE compounds with significant bioactivity. For example, dysidine, a sesquiterpene quinone, extracted from the sponge *Dysidea villosa* has entered preclinical trials for the treatment of diabetes [11].

Plant sterols have attracted recent interest due to their beneficial effects on human health. The FDA (US Food and Drug Administration) and EFSA (European Food Safety Authority) recommend products enriched with phytosterols as part of a dietary strategy to reduce the risk of coronary heart disease [12]. Recent findings suggest that phytosterols play an important role in improving cognition in AD patients by modulating molecular processes involved in AD, such as APP processing and amyloid beta plaque formation both in vitro and in vivo [13,14]. Phytosterols are able to cross the blood brain barrier and accumulate in the membranes of brain cells. Thus, in addition to their role in the pathophysiology of AD, lowered levels of phytosterols function as biomarkers for early AD [15]. Recent studies have reported on AChE inhibitory activity of methanol extracts from several South African seaweeds *(Caulerpa racemose*, *Codium capitatum*, *Ulva fasciata*, *Halimeda cuneate*, *Amphiora ephedraea*, *Amphiora bowerbankii*, *Dictyota humifusa*, *Hypnea valentiae*, *Padina gymnospora*, *Ulva reticulate*, and *Gracilaria edulis*) [16]. Using methanol and ethanol solvents leads to extracts rich in polar flavonoids, highly oxygenated and polar triterpenes, triterpenoids, and sterol glycosides. However, there are limited studies on the neuroprotective activities of phytosterols from marine algae. Ethyl acetate (EtOAc) soluble fractions, obtained from the ethanolic extract of *Ecklonia stolonifera*, resulted in the isolation of the sterols and phlorotannins with acetylcholine esterase inhibitory activity [17,18]. In continuation of our previous studies, we wanted to further investigate marine algae for their neuroprotective activities in terms of acetylcholine inhibition.

The present study investigated 19 marine macroalgae and one seagrass with the aim to evaluate their antioxidant activity, α-amylase inhibitory activity, AChE activity, and their suitability as an effective functional food. High-performance thin-layer chromatography (HPTLC) hyphenated with microchemical (DPPH free radical) and biochemical (α-amylase and AChE enzymatic) derivatizations was used for target-directed identification of biologically active molecules separated on chromatographic plates. Enzyme inhibitory assays require special ambient conditions that can be easily achieved with an open chromatographic system, such as planar chromatography. Column chromatography, while providing high separation efficiency, operates with organic solvents that often inactivate biological enzymes. HPTLC is compatible with both enzymatic (biochemical) and cell-based (biological) assays, since the mobile phase can be easily removed after plate development and before bioassay application. Planar layer chromatography ensures further advantages such as minimal sample preparation, as crude extracts can be applied directly to plates (without losing components), and enables parallel profiling of sample extracts in different assays.

## 2. Results and Discussion

### 2.1. Plate Derivatization

**Figure 1:**
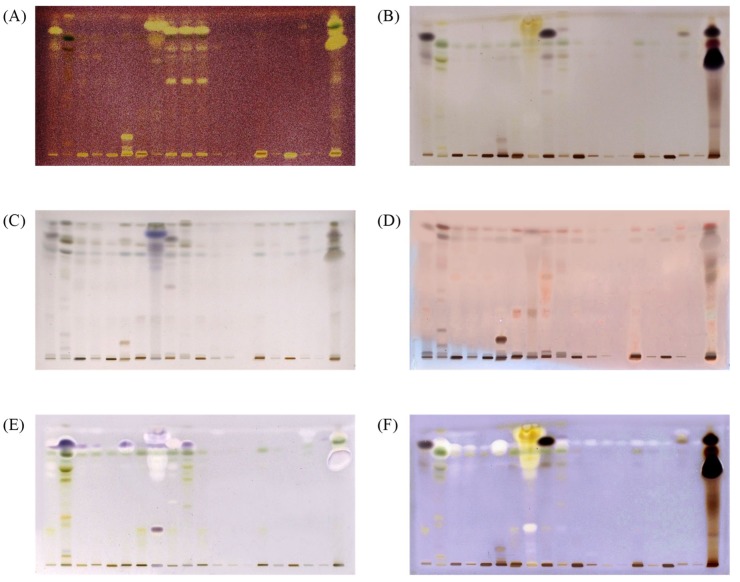
HPTLC chromatograms of extracts under white light after derivatization with DPPH• (**A**), under white light after derivatization with Fast Blue B (**B**), under white light after derivatization with p-anisaldehyde/sulfuric acid (**C**), under 366 nm after derivatization with p-anisaldehyde/sulfuric acid (**D**), under white light after α-amylase inhibitory assay (**E**) under white light after the AChE inhibitory assay (**F**). Mobile phase; *n*-hexane: ethyl acetate: acetic acid in a ratio of 20:9:1 (*v*/*v*/*v*). Tracks 1–20 are corresponding 1–20 algae extracts in ethyl acetate; applied 20 µL per band.

The dried chromatographic plates were subjected to non-targeted chemical derivatizations with p-anisaldehyde and Fast Blue B, and targeted assays using an antioxidant DPPH• free radical, assay and enzymatic assays with α-amylase and AChE. Polyphenolic compounds such as carotenoids, flavonoids, and phenolic acids, are considered to be major contributors to the antioxidant activity in marine algae due to their redox properties. 2,2-Diphenyl-1-picrylhydrazyl free radical (DPPH•) assay is the most commonly used antioxidant assay [9,19]. DPPH• is a deep purple-colored free radical, stable at room temperature due to delocalisation of the spare electron over the whole molecule. Reduction of DPPH• to its non-radical form in the presence of an antioxidant molecule results in a color change to pale yellow [20]. Therefore, after derivatization with DPPH•, compounds with radical-scavenging activity are visualized as bright yellow zones against a purple background on the chromatographic plate (Figure 1A).

After derivatization with p-anisaldehyde reagent, all samples revealed additional bands in the upper part of the chromatograms. p-Anisaldehyde/sulfuric acid is a universal reagent for natural products. However, based on the developed color, secondary metabolites may be differentiated into different classes of compounds [21]. The use of p-anisaldehyde/sulfuric acid as a staining reagent is more informative, because it reveals specific colours for monoterpenes, triterpenes and steroids. In this work, the p-anisaldehyde/sulfuric acid reagent was used to detect the presence of terpenoids/steroids and total terpenoids/steroids expressed in β-sitosterol equivalents.

In vitro α-amylase inhibitory activity was evaluated using a starch test and iodine solution as an indicator. Starch that is not hydrolyzed by the α-amylase, due to enzyme inhibition by the compounds present in the sample, produces dark-blue zones on the TLC plate in the presence of iodine. The inhibition of α-amylase, as purple bands, was observed at an *R_f_* value of 0.85 ± 0.02 (Figure 1F) on the chromatographic plates. Relative α-amylase inhibitory activity in the samples was expressed in acarbose equivalents by comparing the peak areas of blue bands at *R_f_* = 0.85 ± 0.02 with the areas of the blue bands of acarbose standard.

AChE inhibitory activity was detected using the method developed by Hage and Morlock [1]. The α-naphthyl acetate is converted into α-naphthol by AChE, and forms a purple diazo dye complex with Fast Blue B. If AChE is inhibited, a white color is observed at the zones. The results from the present study showed that the marine algae samples contain AChE inhibitory compounds, with a number of white bands observed on the plates (Figure 1). Table 5 shows the total AChE inhibition of all tested extracts expressed in donepezil equivalents.

Free radical scavenging activities were expressed as mg equivalents of gallic acid/band by using a standard curve of gallic acid, α-amylase inhibitory activities in terms of equivalents to mg acarbose mg/band by using a standard curve of acarbose, AChE inhibitory activities in donepezil equivalents, and phytosterol (terpenoid/steroid) content expressed in β-sitosterol equivalents. Standard curves for gallic acid, acarbose, donepezil and β-sitosterol were constructed by plotting the range of applied amounts versus corresponding band areas in pixels (Table 1). The linearity of the method for gallic acid with DPPH• was established to be in the range of 0.05–0.50 µg/band. For β-sitosterol after derivatization with p-anisaldehyde/sulfuric acid, it was in the range of 0.5–5.0 µg/band, for acarbose using the starch-iodine α-amylase inhibitory assay it was in the range of 100–2200 µg/band, while for donepezil using the AChE inhibitory assay it was in the range of 50–600 µg/ band.

### 2.2. Method Validation

Good repeatability of the methods was confirmed by calculating the relative standard deviation (RSD) for repeated measurements at three different concentrations (low, medium and high) of standards within the linear range (Table 2). Averaged RSD values were 5.37, 4.64, 6.55 and 7.03% for acarbose (starch-iodine α-amylase assay), gallic acid (DPPH• assay), β-sitosterol (p-anisaldehyde/sulfuric acid assay), and donepezil (AChE assay), respectively. The relatively low values for RSD indicates good precision of the method allowing quantification of α-amylase inhibitors, antioxidants and phytosterols. Analytical sensitivity of the method was determined by calculating LOD and LOQ as response of 3 and 10 times to the noise. LOD and LOQ of 20 and 60 ng for gallic acid with DPPH•, 0.5 and 1.6 μg for beta-sitosterol with p-anisaldehyde in sulfuric acid, 52 and 173 μg for acarbose in an α-amylase inhibition assay, and 29 and 96 μg for donepezil in AChE inhibition assay were determined.

### 2.3. AChE and α-Amylase Inhibitory Activity

The activity of individual components in the ethyl acetate extracts was detected in situ via α-amylase inhibitory, AChE inhibitory, or an antioxidant assay on the chromatographic plate. The α-amylase inhibiting activity of the marine algae extracts were tested based on the method previously reported by our group [10].

**Figure 2:**
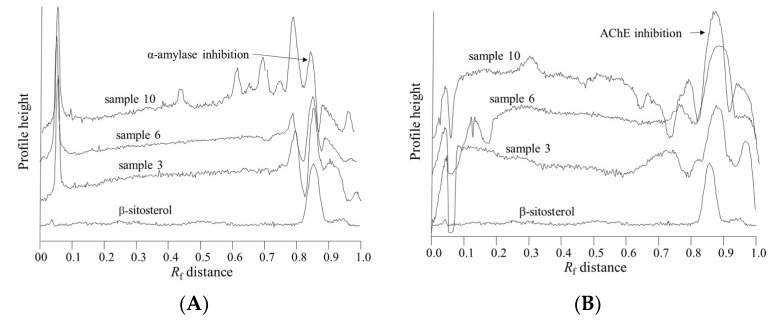
Superimposed β-sitosterol chromatogram obtained after derivatization with anisaldehyde (below) with chromatograms of samples 3, 6 and 10 obtained after (**A**) α-amylase assay and (**B**) acetylcholine esterase assay.

Samples 2, 6, 8 and 10 showed strong inhibition of α-amylase and AChE, together with strong antioxidant properties (Figure 1 and Table 3). The results of in vitro α-amylase inhibitory tests for these samples revealed a more potent inhibition of the enzyme compared to acarbose as a positive control. The highest inhibitory activities for samples 2, 10, 6 and 8 were found to be more than 140, 40, 90 and 80 times more potent than the same amount of acarbose. As seen in Figure 2, our results suggest that α-amylase and AChE inhibitory activities are associated with the presence of phytosterols. In this work, this was confirmed by the colored bands observed on the chromatographic plates obtained after derivatization with p-anisaldehyde/sulfuric acid.

### 2.4. Terpene and Phytosterol Compounds

In order to determine the chemical nature of the compounds with α-amylase inhibitory activity, post chromatographic derivatization with p-anisaldehyde in sulfuric acid was used. p-Anisaldehyde/sulfuric is a universal reagent commonly used for detection of terpenes, carbohydrates and steroids based on color differentiation. Developed and dried chromatograms were sprayed with p-anisaldehyde/sulfuric acid reagent and heated at 100 °C for 10 min. After derivatization and heating, spots of separated compounds exhibited a range of colors from violet, blue, red, gray to green spots. The colors developed after derivatization are related to the chemical type of the compound. It is suitable for the detection of a range of nucleophilic compounds, such as sugars, steroids, terpenes and amines, or compounds bearing these functionalities. A violet color indicates phenolic molecules, a blue/red color is characteristic for amines, aldehydes, ketones, carbohydrates and esters (e.g., alkylphthalates), while a green color indicates allylic alcohols [22].

Monoterpenes, triterpenes and steroids usually appear as blue, purple and gray spots [23]. Derivatization using p-anisaldehyde/sulfuric acid creates brown spots for diterpenes [22], while triterpenes produce a blue violet color under visible/white light, and a reddish or blue color under UV at 366 nm [24,25]. β-Sitosterol (a triterpenoid) was visualized as a blue-violet band under visible light (Figure 1B) or as a red band under 366 nm (*R_f_* = 0.85 ± 0.02).

The colored band obtained after derivatization with p-anisaldehyde/sulfuric indicates that compound responsible for α-amylase inhibition is a terpenoid. The areas of the bands at *R_f_* value of 0.85 ± 0.02, after derivatization with p-anisaldehyde, were highly correlated (*R*^2^ = 0.75) with the blue zones in the α-amylase inhibitory assay, and are related to the bright zones (*R_f_* = 0.87± 0.02) in the AChE inhibitory assay (*R*^2^ = 0.40). The position of the peak, i.e., *R_f_* value, was very close to the *R_f_* value for the β-sitosterol, indicating that plant sterols are responsible for the α-amylase and acetylcholine esterase inhibition (Figure 2). Furthermore, the compound responsible for α-amylase inhibitory activity did not exhibit antioxidant activity in the DPPH• assay (Figure 1).

A potent in vitro alpha-amylase inhibition of the methanolic extract from brown algae *Sargassum glaucescens* has been previously related to phytosterols. Stigmasterol and β-sitosterol were identified by spectroscopic methods, among other sterols, in the extract [26]. Inhibition of α-amylase activity intervenes with metabolism of starch, which forms the main source of nutrition for organisms feeding on plants. Therefore, amylase inhibitors serve as defence compounds in plants [27].

Ethanolic extracts from 27 Korean seaweeds were screened for cholinesterase inhibitory activity. The ethanolic extracts of *Ecklonia stolonifera* and *Ecklonia cava* showed measureable AChEs inhibitory activities. Although various phlorotannins and sterols were isolated from the extracts, phlorotannins were considered to be more potent ChE inhibitors and selected for further studies [28]. Higher solubility of phlorotannins and lower solubility of sterols in methanol and ethanol could lead to this conclusion. For example, solubility of β-sitosterol in different solvents is in the following order: methanol < n-hexane < ethanol < acetone < ethyl acetate [18]. Thus, extraction with ethyl acetate would lead to extracts rich in phytosterols. High in vitro acetylcholinesterase inhibitory activity of β-sitosterol from Salvia root has also been reported. The experimental results were further confirmed by docking analysis [29].

**Figure 3:**
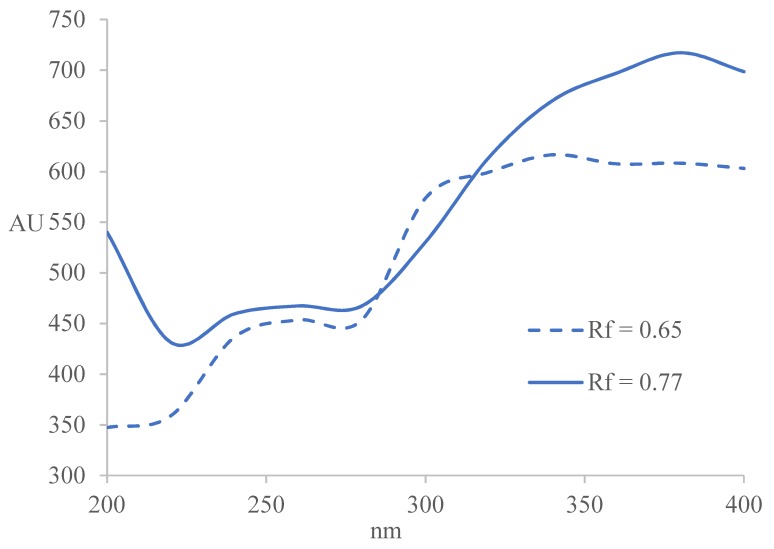
Constructed absorption curves for the compounds in sample extract 2 with *R_f_* = 0.65 ± 0.02 and *R_f_* = 0.77 ± 0.02.

Absorption curves for the compounds in sample extract 2 with *R_f_* = 0.65 ± 0.02 and *R_f_* = 0.77 ± 0.02 were constructed from the multiwavelength densitometric scans from 200–400 nm, with wavelength increment of 20 nm (Figure 3). Sample 2 was selected due to its highest α-amylase and moderate AChE inhibitory activities. The UV absorption spectra of both compounds exhibit two absorption maxima at the following wavelengths: compound with *R_f_* = 0.65 ± 0.02 has absorption maxima at approximately 260 nm and 340–360 nm and compound with *R_f_* = 0.77 ± 0.02 at 260 nm and around 380 nm. Absorption maximum at 260 nm originate from an aromatic group and is characteristic of a p-benzoquinone. Its reduced form displays a characteristic absorption maximum at 340–360 nm.

### 2.5. Flavonoid Compounds

The UV-Vis spectra of flavonoids show strong absorption at the following wavelengths: flavones and biflavones (310–350 nm) and (250–280 nm); isoflavones (310–330 nm) and (245–275 nm); flavonols (350–385 nm) and (250–280 nm); flavanones (310–330 nm) and (275–295 nm); chalcones (365–390 nm) and (240–260 nm); and anthocyanins (465–560 nm) and (265–275 nm) [30,31]. Therefore, the compound at *R_f_* = 0.65 ± 0.02 could be a flavone, while the compound at *R_f_* = 0.77 ± 0.02 is most likely a terpenoid.

### 2.6. Alkylresorcinol Compounds

**Figure 4:**
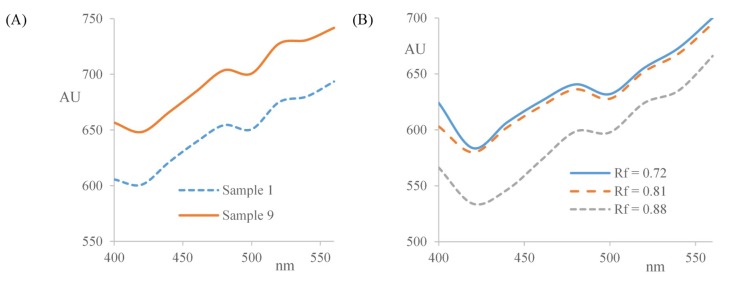
Constructed absorpion curves for: (**A**) the compound in chromatogram at *R_f_* = 0.88 in samples 1 and 9; and (**B**) the compounds at *R_f_* = 0.72, 0.81 and 0.88 in sample 20.

Dark purple bands have been observed after derivatization with fast blue B in samples 1, 9, and especially in sample 20 [32]. The reaction with Fast Blue B salt is highly specific for phenolic lipids. Phenolic lipids are long chain alkyl resorcinol derivatives with an odd numbered alkyl chain at position 5 of the benzene ring (5-n-alkyl derivatives of resorcinol). This structure is responsible for their amphiphilic properties. The side chain is usually saturated, but unsaturated and oxygenated chain analogues have also been reported [33,34,35,36]. According to Kozubek and Tyman [37], there are only two fractions of phenolic lipids that produce violet color products with Fast Blue B, alk(en)ylresorcinols and ketoalk(en)ylresorcinols. Other phenolic compounds produce different color reactions with Fast Blue B salt (e.g., red-violet, weak reddish-pink, brown-reddish violet, yellow orange). Alkylresorcinols form colored azo-products with Fast Blue salt B under alkaline conditions. The chemical nature of the colored products was further investigated by constructing the absorption curves for the purple bands at *R_f_* = 0.88 ± 0.05 for samples 1 and 9, and at *R_f_* = 0.72 ± 0.01, 0.81 ± 0.01 and 0.88 ± 0.01 for sample extract 20, from the multiwavelength densitometric scans in the range 400–560 nm, with wavelength increments of 20 nm (Figure 4). The colored products exhibit two absorption maxima, a peak at 480 nm and a shoulder peak at approximately 520 nm. UV-Vis spectra of colored derivatives of alkylresorcinols with Fast Blue B reagent in methanol have been reported to have a maximum at 480 nm and 520 nm [32,38]. These results confirm the presence of alkylresorcinols in algae sample extracts 1, 9, and 20. These bands were also observed to have significant antioxidant activity with bright bands observed at the same *R_f_* values in the DPPH• assay.

Alkylresorcinols are found in higher plants, algae, mosses, fungi and bacteria [39,40]. Interest in these phenolic lipids is growing. They appear to have a stronger antioxidant effect than resorcinol, indicating the importance of the presence of the lipophilic alkyl chain on alkylresorcinols [41]. In vitro studies have demonstrated that dietary alkylresorcinols show anticancer activities [42].

## 3. Materials and Methods

### 3.1. Chemicals Used

All solvents and chemicals used were of analytical reagent grade. 2,2-Di(4-tert-octylphenyl)-1-picrylhydrazyl (DPPH•) free radical, iron (III) chloride (97%), gallic acid (97%), β-sitosterol (95%), and Gram’s iodine solution were purchased from Sigma-Aldrich (Munich, Germany). α-Amylase from *Bacillus licheniformis* liquid (Cat. No. A4862), AChE from Electrophorus electricus, Fast Blue B Salt, and soluble starch were obtained from Sigma-Aldrich (Munich, Germany). Ethyl acetate, ethanol, ferric chloride (97%), methanol, sodium dihydrogen phosphate (NaH_2_PO_4_) and disodium phosphate (Na_2_HPO_4_) were obtained from Merck (Darmstadt, Germany). Acarbose was from Bayer (Leverkusen, Germany), n-hexane from BDH (Poole, England), p-anisaldehyde from ACROS organics (New Jersey, USA), and donepezil from Cayman chemicals (Ann Arbor, MI, USA). Bovine serum albumin (BSA) was purchased from PAA Laboratories GmH (Haidmannweg, Austria). Tris(hydroxymethyl)aminomethane hydrochloride (Tris HCl) buffer solution was obtained from Calbiochem (San Diego, CA, USA), and Milli-Q water (Millipore^®^, Merck, Darmstadt, Germany) was used to prepare all solutions. All chromatographic separations were performed on 20 × 10 cm normal phase Silica gel 60 F254 HPTLC glass plates (Merck, Darmstadt, Germany).

### 3.2. Sample Collection and Extraction

Twenty fresh marine samples (19 algae and one seagrass) (Table 4) were collected from Torquay beach (Victoria, Australia) and transported in cooled insulated containers to our laboratory. Within six hours of collection, algae samples were thoroughly rinsed three times with filtered seawater, photographed, divided into 50–200 g portions, frozen, and freeze dried using a freeze dryer (Dynavac, FD12, Belmont, Australia). Freeze dried samples were ground to a fine powder. Approximately 5 g of finely ground sample was extracted 5 times by vigorous shaking for 15 min with 50 mL of ethyl acetate, in sealed glass stoppered conical flasks and filtered. The combined extracts were evaporated to approximately 6 mL under vacuum, transferred to a 10 mL volumetric flask, and adjusted to volume with ethyl acetate. All extracts were stored at 4 °C to minimize degradation.

### 3.3. High Performance Thin Layer Chromatography (HPTLC)

Plates were pre-washed before use with a blank run of methanol and activated by drying in an oven at 100 °C for 30 min. Sample extracts and standards were sprayed onto the HPTLC plates as 6 mm bands using a TLC sampler (Linomat IV, CAMAG, Muttenz, Switzerland), 8 mm from the lower edge, with 14 mm distance from each side, and a minimum distance of 2 mm between each tracks.

Separation was performed with n-hexane, ethyl acetate, acetic acid (15:9:1) in an Automated Multiple Development Chamber (AMD2, CAMAG) up to a migration distance of 80 mm. Densitometric analysis was performed using the TLC Scanner 4 (CAMAG, Muttenz, Switzerland) controlled by winCATS Planar Chromatography manager software version 1.4.6 (CAMAG, Muttenz, Switzerland). HPTLC data were processed and evaluated using the digital image analysis software Sorbfil TLC Videodensitometer (Sorbpolymer, Krasnodar, Russia).

### 3.4. Post Chromatographic Derivatization

A 0.2% (*w*/*v*) DPPH• solution was prepared in methanol (Merck, Darmstadt, Germany), stored at 2–8 °C, and protected from light. Neutralized ferric chloride solution was prepared by adding dilute sodium hydroxide solution to freshly prepared 2% (*w*/*v*) ferric chloride solution in methanol, drop by drop until some ferric hydroxide just precipitates. The solution was then filtered to remove precipitate and the clear filtrate was used for derivatization [43]. The p-anisaldehyde reagent solution was freshly prepared by combining 1 mL p-anisaldehyde with a refrigerated solution of glacial acetic acid/concentrated sulphuric acid in methanol in the ratio of 0.5:50:1. The colorless solution was stored at 2–8 °C. (Note: if a pink discoloration develops, the reagent must be discarded.) The plate neutraliser buffer solution was prepared by adding 1 M NaH_2_PO_4_ dropwise into 100 mL of 1 M Na_2_HPO_4_ until a pH of 7.5 is reached. The Fast Blue B spray reagent 0.1% (*w*/*v*) was prepared by dissolving Fast Blue B in 70% (*v*/*v*) ethanol. A 1% (*w*/*v*) α-amylase solution was prepared by diluting approximately 1.25 mL (1 g) of α-amylase with distilled water to a total volume of 100 mL [44]. The enzyme stock solution was stored at 2–8 °C.

A 2% *w*/*v* starch solution was prepared by adding a smooth paste of soluble starch into boiling water and stirring until all of the starch is dissolved (the resulting solution may be cloudy). Starch is a heteropolysaccharide, composed of amylopectin (70–80%) and amylose (20–30%) polysaccharides [45]. Amylose forms inclusion complexes with iodine once the insoluble starch granular structure is destroyed by heating it in water. When the amylose is molecularly dispersed in water, it forms colored complexes with iodine.

A 0.003% *w*/*v* AChE solution was prepared by dissolving 3 mg AChE in 100 mL of Tris hydrochloride buffer (~pH 7.5) with a 100 mg of BSA. The AChE assay reagent was freshly prepared by adding 42 mg 1-naphthyl acetate and 84 mg of Fast Blue B Salt into a mixture of 50 mL ethanol and 100 mL of Milli-Q purified water. The plate neutraliser buffer solution was prepared by adding 1 M NaH_2_PO_4_ dropwise into 100 mL of 1 M Na_2_HPO_4_ until the pH reached 7.5.

Derivatization was achieved by immersing plates in the appropriate derivatization solution using the Chromatogram Immersion Device (CAMAG, Muttenz, Switzerland). Antioxidant activity was determined by first dipping plates in a methanolic DPPH• solution (0.2% *w*/*v*) and then storing them in the dark for 30 min. Antioxidants were visualized as bright zones against a purple background. Steroids and terpenes were detected after derivatization with freshly prepared p-anisaldehyde/sulfuric acid solution and then heating the derivatized plates at 105 °C until maximum visualization of spots (approximately for 10 min). Derivatization with Fast Blue B was used for detection of phenols, with alkylresorcinols detected as dark purple zones on an almost colorless background. Plate images of all plates were recorded using the TLC Visualizer Documentation System (CAMAG, Muttenz, Switzerland) equipped with a 12-bit charged couple device (CCD) digital camera and winCATS software (CAMAG, Muttenz, Switzerland), under white light illumination in the reflectance mode.

### 3.5. HPTLC-EDA

α-Amylase inhibition assays were carried out according to our previously reported method [10]. Developed plates were first dried to remove any traces of mobile phase and then dipped into an α-amylase solution. They were then incubated for 30 min at 37 °C, dipped into the substrate solution (2% *w*/*v* starch), and incubated for another 10–20 min in order for the enzyme substrate reaction to go to completion. Plates were then dipped in Gram’s Iodine solution and plate images recorded. Observed dark blue zones against a white background indicates α-amylase inhibition activity.

Acetylcholinesterase (AChE) inhibitory activity was detected using the method developed by Hage and Morlock [1]. A developed plate was first neutralized by dipping into the plate neutraliser buffer solution 6 times for 1 sec at an immersion speed of 5cm/s using the Chromatogram Immersion Device (CAMAG, Muttenz, Switzerland). It was then incubated in a humidity chamber at 37 °C for 30 min, dipped into a solution of the substrate (α-naphthyl acetate), and then into the dye reagent (Fast Blue Salt B). Bright zones against a purple background on the plate indicate AChE inhibitory activity.

### 3.6. Method Validation

The methods for acarbose, gallic acid and β-sitosterol determinations were validated according to the International Conference on Harmonization (ICH) guidelines [46]. The working range was assessed by plotting chromatographic peak areas versus standard concentration (µg/band). Linear ranges were established using the least squared regression analysis in Excel^®^. Repeatability was assessed by applying three repetitions of each standard at three concentration levels (low, medium and high) within the calibration curve. Variance between repetitions was expressed as a relative standard deviation (%RSD). The sensitivity of measurements was estimated in terms of the limit of quantitation (LOQ) and limit of detection (LOD). LOQ and LOD were calculated by the use of following equation:

LOD = 3 × *Sd*/*B*

LOQ = 10 × *Sd*/*B*

where *Sd* is the standard deviation of the peak areas of the standards (*n* = 3), taken as a measure of noise, and *B* is the slope of the corresponding calibration curve.

## 4. Conclusions

This study demonstrates the combined power of biochemical and chemical HPTLC fingerprints in effect-directed analysis. HPTLC is a powerful tool in drug discovery, especially in target-directed identification of biologically active molecules in complex samples, when combined with chemical and biochemical assays. HPTLC hyphenations enabled the screening for bioactive (antioxidant, antidiabetic and ChE inhibiting) compounds from ethyl acetate extracts of 19 marine algae samples and one sea grass collected from Torquay beach (Victoria, Australia). Significant α-amylase and AChE inhibitory activities were observed in samples 2, 6, 8 and 10. Samples 1, 8, and especially sample 20, were found to contain phenolic lipids (alkyl resorcinol derivatives) with significant antioxidant activities. These marine algae contain a number of compounds with antidiabetic and neuroprotective activity that may prove useful for the preparation and development of novel functional ingredients for pharmaceuticals and functional foods in the treatment and/or prevention of diabetes and neurodegenerative diseases. For example, the green alga *Codium fragile* (sample 2) has been shown to possess significant α-amylase and AChE inhibitory activities in vitro. *C. fragile* is a known invader in marine ecosystems around the world, with negative economic effects on other aquaculture species. Thus, industrial exploitation of this raw material could assist in controlling its proliferation. Sample 20 was found to contain high amounts of phenolic lipids. Omega-3 polyunsaturated fatty acids from fish oils promote well-established health and antiaging benefits that justify their use as functional ingredients in dietary supplements, healthy foods, and nutraceutical products. However, the practical use of such lipids as food ingredients is limited due to their high susceptibility to oxidation and loss of nutritional value. Phenolic lipids are a good solution for these problems. There is interest in the use of edible marine macroalga for the development of low-cost, highly nutritive diets for human and animal nutrition. Nevertheless, there is an increasing need to extend the research in this area and further investigate the content of the sterols present in these algae.

## Figures and Tables

**Table 1:** Regression data.

Standard	Method	Regression Equations (Linear and Nonlinear)	*R* ^2^	Range (μg/band)	LOD (μg)	LOQ (μg)
Acarbose	Starch/iodine test	*y =* 91.23*x +* 4125.2 *y = −*0.0117*x^2^ +* 114.47*x −* 1934.5	0.969 0.988	100–2200 100–4000	52	173
Gallic acid	DPPH•	*y* = 780150*x* + 66380 *y* = *−*487389*x*^2^ + 10*^−^*^6^*x* + 39382	0.939 0.983	0.05–0.50 0.05–1.5	0.02	0.06
β-sitosterol	p-Anisaldehyde/sulfuric acid	*y =* 14645*x +* 16883 *y = −*349.43*x^2^ +* 14185*x +* 19341	0.963 0.979	0.5–5.0 0.5–12.0	0.5	1.6
Donepezil	Ellman’s reaction	*y =* 202.15*x +* 14999 *y = −*0.1849*x^2^ +* 302.33*x +* 4994.8	0.970 0.967	50–600 50–1000	29	96

**Table 2:** Method accuracy in terms of relative standard deviations for replicate measurements (*n* = 5) at three different levels.

Acarbose		Gallic Acid		Donepezil		β-Sitosterol	
Applied (µg)	RDS (%)	Applied (µg)	RDS (%)	Applied (µg)	RDS (%)	Applied (µg)	RDS (%)
200	7.20	0.05	8.17	50	6.96	1.00	7.06
400	2.98	0.10	3.16	500	6.02	2.00	7.88
900	5.93	0.20	2.59	1000	8.11	4.00	4.72
average	5.37		4.64		7.03		6.55

**Table 3:** α-Amylase inhibition, phytosterol content, antioxidant activity and AChE inhibition in ethyl acetate extracts.

Sample	AE µg/Band	SE µg/Band	GAE ng/Band	DE µg/Band
1	204	45	540	0
2	3927	61	30	1072
3	644	26	20	276
4	411	20	40	255
5	299	14	20	116
6	1451	35	240	1521
7	279	20	20	267
8	2606	105	210	2515
9	501	30	290	51
10	1925	32	310	819
11	51	14	440	17
12	75	5	60	185
13	20	1	*	32
14	14	*	*	12
15	301	24	140	170
16	44	5	10	91
17	11	14	30	97
18	91	11	90	194
19	205	2	30	378
20	307	150	1410	*
*R* ^2^	α-Amylase inhibition	0.4	NC	0.8
	Phytosterol content		0.8	NC
	Antioxidant activity			NC

* Below limit of quantification; AE = acarbose equivalent; SE= β-sitosterol equivalents; GAE = gallic acid equivalents; DE = donepezil equivalents; NC = no correlation.

**Table 4:** Marine species used in this work.

Sample Number	Species	Type of Marine Species
1	*Cystophora pectinata* (Greville & C.Agardh ex Sonder) J.Agardh	brown algae
2	*Codium fragile subsp. tasmanicum* (J.Agardh) P.C.Silva	green algae
3	*Pyllospora comoasa* (Labillardière) C.Agardh	brown algae
4	*Scytothalia dorycarpa* (Turner) Greville	brown algae
5	*Carpoglossum confluens (R. Brown ex Turner) Kützing*	brown algae
6	*Ecklonia radiata (C.Agardh) J.Agardh*	brown algae
7	*Sargassum lacerifolium* (Turner) C.Agardh	brown algae
8	*Perithalia caudata* (Labillardière) Womersley	brown algae
9	*Cystophora harveyi* Womersley	brown algae
10	*Amphibolis antarctica* (Labillardière) Sonder & Ascherson ex Ascherson	seagrass
11	*Scytothalia dorycarpa* (Turner) Greville	brown algae
12	*Hypnea valida* J.Agardh	brown algae
13	*Austrophyllis harveyana* (J.Agardh) Womersley & Norris	red algae
14	*Plocamium dilatatum* J.Agardh	red algae
15	*Cystophora monilifera* J.Agardh	brown algae
16	*Rhodophyllis membaneacea* (Harvey) Hooker & Harvey ex Harvey	red algae
17	*Hormosira banksii* (Turner) Decaisne	brown algae
18	*Myriodesma integrifolium* Harvey	brown algae
19	Epiphytic algae sp.	brown algae
20	*Cystophora subfarcinata* (Mertens) J.Agardh	brown algae
